# Conceiving one‘s national group as transgenerational: Effects on attitudes towards ‘foreign’ and diaspora migrants

**DOI:** 10.1371/journal.pone.0230303

**Published:** 2020-04-02

**Authors:** Klaus Boehnke, David Schiefer, Marieke Christina van Egmond, Katja Hanke, Yechiel Klar, Sonia Roccas

**Affiliations:** 1 Jacobs University Bremen, Bremen, Germany; 2 National Research University Higher School of Economics, Moscow, Russian Federation; 3 German Center for Integration and Migration Research (DeZIM), Berlin, Germany; 4 University of Hagen, Hagen, Germany; 5 University of Applied Management Studies, Mannheim, Germany; 6 GESIS-Leibniz Institute for the Social Sciences, Mannheim, Germany; 7 Tel Aviv University, Tel Aviv, Israel; 8 The Open University of Israel, Raʿanana, Israel; Valparaiso University, UNITED STATES

## Abstract

The current paper presents three studies, which suggest that perceiving one’s nation as transgenerational (*TG*) is related to a differentiation in the evaluation of ethnically German diaspora migrants and ethnically non-German (‘foreign’) migrants. First, we find that unlike ‘classical’ concepts such as right-wing authoritarianism (RWA), social dominance orientation (SDO), and hierarchic self-interest (HSI), *TG* explains differences in derogatory sentiments expressed towards diaspora and ‘foreign’ migrants. Second, *TG* is differentially related to positive emotions and behavioral intentions expressed towards these two groups of migrants. Lastly, results indicate that people who perceive the ingroup as *TG* require ‘foreign’ migrants to fulfill more criteria that make them eligible for citizenship and are thereby more exclusionist than people who include only the current generation into their concept of national identity. The social implications of these findings in face of the so-called refugee crisis in Germany and the wider European Union are discussed.

## Introduction: The temporal dimension of group identification

Social psychological approaches to understand the nature, conditions, and consequences of identifying with groups have for a long time primarily viewed the group as a current structure, e.g., individuals currently belonging to different football clubs or people belonging to particular nations. With regard to the identification with groups such as the nation, the inclusion of a temporal dimension can, however, be of particular relevance [1]. Such temporal dimensions of group identification have long been the domain of historians, philosophers, cultural anthropologists, and political science scholars, in particular with regard to the concept of nationalism [e.g., 2–4]. Only recently, recognition of the importance of the temporal dimension in the representation of groups has also been growing among social psychologists [5–8]. Specifically, Sani et al. [6] coined the term “perceived collective continuity” to denote that groups are often perceived as entities that move through time. As already noted early on by Radcliffe-Brown [9], group membership refers not only to "the members now living but also to those who have died and to those who are not yet born [9: p.144), an assertion that had, however, been ignored by social psychologists during the last 70 years.

The inclusion of the temporal dimension breaks with the classical conceptualization of national group identification prevalent in social psychology: When people refer to their national or ethnic group, they do not necessarily represent it as merely a social structure consisting of contemporary group members. In fact, it is an inherent component of the concept of the ‘nation’ that both its life-span and existence extend beyond its current cohort of members into the past as well as the future. The idea of a common history and ancestry was among the founding principles of the ideology of nationalism and the creation of nationhood [10–12] This may also explain, for example, why contemporary Germans struggle with their sense of national identity since it encompasses the unmatched crimes against humanity that were committed by Germans of previous generations during the Nazi era [13].

Kahn, Klar, and Roccas [1] have presented a social psychological approach to the concept of group identification that explicitly incorporates the temporal dimension. According to this approach, viewing one´s group as transgenerational is thought to be associated with a sense of belonging and loyalty that not only lies with those who are current members of the group, but with all the generations of the group that ever have existed and ever will exist. The current generation is perceived as the link in the transgenerational chain that constitutes the group rather than being the begin-all and end-all of the group.

Kahn et al.’s [1] approach assumes that the extent to which a person views the ingroup as spanning across past, current and future generations (as opposed to just encompassing the current generation) is related to other psychological phenomena such as the acceptance of endured ingroup sacrifices.

## A transgenerational view on one’s own group and the evaluation of outgroups

The current paper further examines the temporal dimension of ingroup identity. In particular, we will show that an individual’s representation of the national ingroup as not only including the current generation but also the past as well as future generations is related to the person’s evaluation of different outgroups. Following Kahn et al. [1], we thus distinguish between perceiving the group as ***t***rans***g***enerational (*TG*) and perceiving it as ***i***ntra***g***enerational (*IG*). We test (and confirm) the assumption that viewing one’s nation as *TG* leads to more negative evaluations of what we term ‘foreign’ migrants but less so with regard to what we term diaspora migrants (see the sections below for more details on these types of migrant groups). The paper furthermore documents that *TG* can explain individuals’ evaluations of outgroups in different ways compared to other theoretical constructs that are known to predict outgroup evaluations.

In the subsequent theoretical sections, we will first provide some theoretical background on the concept of *TG* vis-á-vis other constructs and their relation to outgroup evaluations. We will then theoretically derive hypotheses and document how we tested them empirically.

### Background: The temporal dimension in previous approaches to ingroup-outgroup perceptions

A number of previous conceptualizations of how individuals perceive their national ingroup vis-á-vis national outgroups have to some extent considered temporal aspects of ingroup perceptions. However, these approaches have either done so only indirectly and have provided only scarce empirical evidence of its effects on other psychological phenomena, or they have incorporated the temporal dimension in a different way, emphasizing different aspects. The following two sub-sections elaborate on such constructs and how they differ from *TG*.

#### Nationalism

A most relevant concept that describes the relationship between ingroup attachment and outgroup evaluation is nationalism. In terms of social identity theory [14], nationalism has been conceived as an intergroup differentiation, which is inherently related to ingroup favoritism which may lead to outgroup derogation. Later approaches argued that ingroup favoritism does not necessarily need to be associated with outgroup derogation [15], but that nationalism rather represents the detrimental facet of positive ingroup evaluation since it includes the view that one’s country is superior to others and hence should be dominant [16,17]. These approaches by and large neglected the temporal dimension of national ingroup favoritism and outgroup evaluation. One exception is a study by Mummendey, Klink, and Brown [18], who investigated the effect of ‘patriotic’ and ‘nationalistic’ attitudes on outgroup derogation: They tested whether xenophobic attitudes differ depending on the degree to which individuals favor their national group compared to (1) other countries, (2) to how that country had fared in the past (or might fare in the future), or (3) to a socio-political prototype of an ‘ideal’ society [18]. However, they did not find any empircal evidence that the temporal comparison is differently associated with outgroup rejection than the other comparisons.

Another common distinction made in the nationalism literature is the distinction between ethnic and civic forms of nationalism [19–21]. Civic nationalism refers to defining the national group on the basis of common citizenship and participation in public life. As opposed to that, in ethnic nationalism the national group is defined in terms of ethnicity, which involves ascribed criteria such as ancestry. This definition of the national ingroup also implies that the nation is seen as an entity that has *naturally emerged* (over time) and has manifested politically, rather than as a *politically created* community [20]. Historical analyses have shown that these different conceptualizations of nationalism are associated with the way in which nations have shaped their citizenship policies. In Germany, for example, policies have long been based on the ‘jus sanguinis’ principle, whereby ancestral ethnic ties are the primary basis for citizenship. Only recently, the strict ‘jus sanguinis’ principle has been loosened in favor of the ‘jus soli’ principle for second and third generation immigrants. People of German descent who come to Germany from Eastern Europe and Russia or other states of the former Soviet Union (so called *Aussiedler*, i.e., resettlers) have a constitutionally guaranteed entitlement to immediate German citizenship, whereas for a long time second-generation Turkish immigrants [20] have had an entitlement to German citizenship only under restricted conditions. In contrast, France’s citizenship policies, e.g., were designed on the basis of the ‘jus soli,’ where citizenship is granted on the basis of birth within a territory.

However, despite the fact that approaches to nationalism as well as citizenship policies have incorporated the temporal dimension in the conceptualization of the national group, empirical evidence that this dimension is associated with the evaluation of outgroups is scarce. Formal citizenship policies, for example, do not easily translate into attitudes, emotions, and behaviors. There is, however, at least rudimentary empirical evidence to suggest that national citizenship policies are related to in- and outgroup evaluations of national group members; people can hold attitudes about their country that are either based on an ethnic or civic representation of their national group, regardless of the official citizenship policy of their country [22].

#### Adjacent group conceptualizations

Several lines of research have started to address the effects of the incorporation of a temporal dimension of group conceptualizations. Although related to each other and the concept of *TG*, several distinctions can be made.

Reicher and Hopkins [5], for example, provide a historical account of how the past shaped collective identity and nationalistic sentiment. Also taking a historical perspective, Liu and Hilton [23] describe how the representation of a nation’s history (the nation’s ‘charter’) can shape group boundaries and define ingroup responsibilities. That may then serve as a justification of a specific political agenda that underscores the exclusionary character of the ‘nation’ vis-à-vis outgroup members. In contrast to *TG*, these two conceptual accounts do exclusively look back in history when delineating the temporal dimension of conceiving one’s nation, without referring to continuity into the future (as the definition of *TG* does).

Another concept, *collective nostalgia*, has been described primarily in terms of individuals’ perceptions of the nation’s positive attributes [24]. In contrast to *TG*, however, which refers to including in the representation of the ingroup all past as well as future generations, collective nostalgia describes how historical sentiments are included in an individual’s national identity [8]. In doing so, it includes an emotional element whereas *TG* solely refers to the perception of the temporal boundaries of the national group. Furthermore, the use of the concept of ‘transgenerational’ implies that it is not merely the past that can play a role for intra- and intergroup phenomena, but also the perception of the continuation of the group into the *future*. Finally, whereas collective nostalgia ‘solely’ deals with the emotional meaning of one’s nation’s attributes, *TG* deals with qualitatively different perceptions of one’s national group: If people perceive their nation as only consisting of currently living group members (i.e., low *TG*), they exhibit a narrow, concrete conceptualization of the group. In contrast, people who have the feeling that their national group extends to the past and the future (i.e., high *TG*), their conceptualization of the national ingroup is more symbolic and broader. The current generation is conceived as the connecting bridge between past and future generations and is thought of as the group’s caretaker. These individual differences in the perceptions of the national group, so the assumption, has an impact on the way people deal with outgroups and intergroup conflicts.

In relation to outgroup evaluations, a third concept, *collective angst* (i.e., concern for the group's future), is of particular significance [25,26]. Jetten and Wohl [27], for example, find that a perceived discontinuity of the group’s past into the present can undermine the vitality the group is perceived to hold in the future. This feeling increases the sense that the current collective identity needs to be preserved, leading to more negative attitudes towards immigrants. This same mental process has also been studied in the context of terror management theory [28]. Collective angst differs from *TG* in that it not so much emphasizes continuity of the nation from past to future, but puts a certain collective emotion in the forefront.

Finally, the concept of *Perceived Collective Continuity* (PCC), which was proposed by Sani et al. [6], captures the extent to which people see the norms and traditions that define their group as having remained similar across the ages. It also encapsulates the degree to which the history of the group is seen as causally connected. PCC consists of two related dimensions of perceived temporal continuity of the group. Namely, perceived cultural continuity refers to the extent to which group norms are seen as transmitted from one generation to another, and perceived historical continuity refers to the extent to which the different ages, periods, and events in the group history are perceived to be causally interconnected [6,29]. Although PCC includes a temporal dimension of group perception, it differs from *TG* by the fact that it does not deal explicitly with the level of inclusiveness in its members’ group perception (i.e., with the question of who is included in the national group and who is not; see Kahn et al. [1]). Rather, it refers to the extent to which group members perceive the culture and history of the group to be closely interconnected across history. This conceptual difference has been confirmed by Kahn et al. [1] who show that in a factor analysis including PCC and *TG* items the two concepts prove to be empirically distinct and that *TG* explains other phenomena such as the acceptance of endured ingroup suffering over and above the effect of PCC.

#### The current paper: TG and differential prediction of outgroup evaluations

A common characteristic of the approaches described in the previous section is that they assume a rather uniform link between national ingroup identification and outgroup evaluations without distinguishing between different kinds of outgroups. In contrast, this paper argues that viewing one’s nation as *TG* can be related to the perception of different national outgroups in different ways. We investigate this argument using the example of attitudes towards two types of immigrant groups living in Germany: On the one hand these are immigrants who came to Germany for work reasons (Turkish ‘guest workers’), on the other hand there is a group that migrated to Germany from Eastern Europe in order to become part of the state on the basis of their ancestral ethnic ties, mainly in the 1980s and early 1990s [30]. The latter are commonly called *Aussiedler* (resettlers), or—if they came after 1992—*Spätaussiedler* (late resettlers). The differentiation between *Aussiedler* and *Spätaussiedler* refers back to a law that restricted the immigration of ethnic Germans at the end of 1992. Compared to the earlier cohorts, *Spätaussiedler* who came during the 1990s were economically disadvantaged due to an average lower German language proficiency and professional trainings that were less adequate for the German labor market [30]. Both *Aussiedler* and *Spätaussiedler* had been part of the German people centuries ago until they emigrated to Eastern Europe [31]. Due to this German ancestry, and in contrast to other immigrant groups, diaspora migrants of either group are guaranteed German citizenship immediately after their arrival by the German constitution, Article 116. Entry into Germany may, however, be restricted by simple law.

It is worth mentioning that such an immigrant group is also present in Israel, where the *TG* concept has been developed originally. Those are Jewish immigrants from Former Soviet Union countries, thus immigrants that are related to the national ingroup via the same religious affiliation. Silbereisen [32] suggested using the overarching term “diaspora migrants” (p. 2) for them. In orthography, it has become customary to capitalize this term for Jews resettling to Israel, whereas for other resettlers the term usually is not capitalized.

Germany has received several peaks of immigration in the post-Second World War period. The first spanned the years between the mid-1960s and the mid-1970s, when millions of so-called *Gastarbeiter* (guest workers) came to West Germany from Southern Europe, the largest contingent originating from Turkey. The second peak came around the fall of the Iron Curtain in the late 1980s, early 1990s, when migrants mostly came from Eastern Europe and the (dissolving) Soviet Union. The third peak emerged in the first half of the 1990s when hundreds of thousands of people fled from various war and crisis regions in the world to Germany (many from the dissolving Yugoslavia) and applied for asylum [33]. The fourth and most recent peak came about during another strong refugee movement, the so-called refugee crisis in 2015 [34].

In the current paper, we test if the perception of one’s nation as transgenerational (*TG*) differentially affects the way individuals react to what we, in the following, label as diaspora migrants vs. ‘foreign’ migrants (exemplified by Turkish former guest workers). We do this in three studies with different experimental designs and different measures of outgroup evaluations. Our hypothesis is that Germans with a transgenerational conception of their nation will exhibit more *negative* reactions to ‘foreign’ migrants than Germans low on *TG*, while they will, in contrast, view diaspora migrants less negatively or even more *positively* compared to Germans low on *TG*. Why should that be the case? Based on the general assumption, that ingroup favoritism goes along with outgroup devaluation [14], the crucial question is whether or not a group is viewed as being an outgroup or part of the ingroup. Individuals who view their nation as spanning across generations are assumed to more likely accept diaspora migrants (who came as immigrants but who had been part of the German people in earlier generations) as ingroup members. In contrast, ‘foreign’ migrants who came during the last decades will be perceived as national outgroups, since they did not belong to the German people in previous generations and thus are viewed more negatively. For individuals who view their nation as only consisting of people currently living in Germany, the differentiation between the two immigrant groups should be less strong, or in other words, they should accept or reject both immigrant groups in similar ways.

In addition to the above described hypothesis, we investigate two additional questions in order to strengthen the plausibility of the *TG* concepts and its relation to outgroup evaluations: In Study 1, we additionally test whether established predictors of outgroup derogation such as Right-Wing Authoritarianism (RWA) [35], Social Dominance Orientation (SDO) [36], and Hierarchic Self-Interest (HSI) [37] are also able to differentiate between attitudes towards different immigrant groups. Our hypothesis is that this is not the case and that only *TG* relates differentially to the perception of different migrant groups (Hypothesis 2). Demonstrating this would be a strong indicator that the temporal dimension of national identity contributes to the understanding of individuals’ outgroup evaluations in a way that other established constructs are not able to. In Study 2, we additionally investigate how *TG* relates to outgroup attitudes in comparison to the general level of identification with the nation. One could argue that *TG* is just another variant for measuring identification with one’s nation and that the temporal dimension does in fact not play an additional role. Our hypothesis is, however, that *TG* predicts the evaluation of immigrants over and above identification with the nation (Hypothesis 3a) and that identification with the nation itself is not able to differentially predict attitudes towards diaspora migrants and ‘foreign’ migrants the way *TG* does (Hypothesis 3b).

Since all three studies compare attitudes towards ethnic German diaspora migrants and Turkish immigrants with regard to their relation to *TG*, a short description of the two target groups seems in place. The societal integration of both groups as well as their acceptance by the non-immigrant majority population differs to some extent but also shows astonishing similarities. Most of the Turkish guest workers came to Germany with a low educational and professional background [38]. They were thought to stay in Germany only temporarily. Hence, there were initially no support systems facilitating their integration, and even though many eventually decided to stay and bring their families, it appeared to be rather difficult for them to reach a sufficient economic and social status in the German society. This societal disadvantage more or less persists until today. In turn, the perception of them ‘not being integrated’ continues to produce hostile attitudes and derogatory narratives among the majority population (partly also due to the association of this group with Islam). By contrast, diaspora migrants have typically been in a more privileged situation because they were being granted German citizenship immediately after their arrival and thus had certain rights and access to support systems that other groups did not immediately have [30]. Furthermore, their education level at the time of arrival was more heterogeneous compared to the rather low level among Turkish guest workers [39,40]. Current statistics show that German diaspora immigrants are somewhat better integrated into the German labor market and education system than are Turkish immigrants [41]. However, the supposition of a smoother societal integration and higher acceptance of diaspora migrants compared to other groups that was put forward by political and civil society agents and also by researchers in the first place held only to a certain extent [30]. Diaspora migrants (especially those who arrived during the 1990s) did face obstacles regarding their access to the labor market, and many entered job segments that were below their qualification levels [30]. In addition, although being of German descent, diaspora migrants are often viewed as ‘Russian immigrants’ by the autochthonous German population and thus also face discrimination [42,30]. Acculturation orientations of both Turkish immigrants and diaspora migrants tend to lean towards a separation orientation (stronger attachment towards the migrant ingroup than towards the German majority population) [43,30], which is partly due to experiences of social exclusion.

## Study 1

The aim of study 1 was twofold: Firstly, it investigates Hypothesis 1, which states that individuals high on *TG* distinguish between diaspora and ‘foreign’ migrants more strongly and exhibit more differentiated reactions to these groups than those low in *TG*. As sketched, the term *diaspora migrants* refers to outgroup members who have an ancestral relationship to Germany (*Aussiedler* and *Spätaussiedler*). ‘*Foreign’ migrants*, on the other hand, refers to migrants who have come to Germany without having ancestral links to the host country (e.g., Turkish workers). In addition, Study 1 tests Hypothesis 2 which states that the assumed differential relationships between *TG* and derogatory outgroup evaluations are found only for *TG*, but *not* for known predictors of strong outgroup derogation, namely RWA, SDO, and HSI.

### Method

#### Participants

Study 1 was conducted with 190 non-immigrant German individuals (54% female, mean age = 49.8, ranging from 14 to 82). The question arises as to whether sample sizes are adequate for the tests undertaken here. Drawing on Westland [44], we can say that when assuming a medium size effect for differences in target assessment (Aussiedler vs. 'foreign' migrants) and a desired power of. 80 and when setting out to only test the intergroup effect, our samples are almost perfectly sized (assessed across all three studies). Had we set out to test a full structural model that includes all predictor variables (TG, RWA, SDO, HSI) in one model (which is, however, not our intention), our sample sizes would be relatively small. Data were collected via an online survey (N = 76) distributed through various web-based forums and online social networks, and in courses of the local adult education center (German *Volkshochschule*, N = 114). It was decided to collect data in this institution because course participants come from a wide range of socio-economic backgrounds. Overall, participants had a high level of education (more than one quarter reported holding a university degree), expressed low levels of religiosity (more than a third declared to ‘not at all’ be religious, whereas only some three percent saw themselves as very religious), and their political preferences clearly leaned towards the left (some 57% saw themselves as left to liberal, 25% saw themselves as moderates, whereas only 18% saw themselves as conservative to right-wing).

#### Measures

Conceptualized as a predictor/independent variable, the perception of the ingroup as *transgenerational* was measured with a four-item version of the original five-item scale developed by Kahn et al. [1]. The original scale comprised a reversely scaled item which, however, lowered the scale’s reliability in the present sample. The four-item version showed satisfactory reliability (α = .73). The four items were: “For me, my national group includes all the generations of group members that ever have and ever will live”; “When I think of my national group, I don't only think of the current generation but also of all the generations of group members of the past”; “When I think of my national group, I don't only think of the current generation but also of all the generations of group members of the future”, and “Members of my national group in every generation share a common base that unite each other across the generations”. Response options ranged from ‘1’ (lowest level of agreement) to ‘7’ (highest level of agreement).

As a second set of predictor/independent variables, explanatory competitors of *TG*, so-to-speak, were included, namely *right-wing authoritarianism* (RWA, 12 items, α = .80), *social dominance orientation* (SDO, 16 items, α = .87), and *hierarchic self-interest* (HSI, 10 items, α = .80), which were assessed using existing German versions available from the literature. (RWA [45]; SDO [46]; HSI [47]).

*Derogation of* diaspora (i.e., *Aussiedler* and *Spätaussiedler*) and ‘foreign’ (i.e., Turkish) migrants was assessed comprehensively using a combination of two types of measures. The first assessed attitudes, tapping into the cognitive aspect of derogation. It comprised two subsets of six items each, developed by the research team. One subset alluded to adult-age diaspora migrants or Turkish migrants, the other to *children of* diaspora migrants or Turkish migrants. The items were: “(Children of) *Aussiedler*/Turkish migrants are a burden to the welfare system,” “(Children of) *Aussiedler*/Turkish migrants contribute to the cultural wealth in Germany,” The German state must facilitate the integration of (Children of) *Aussiedler*/Turkish migrants into the education system in the same way like for every other German,” “(Children of) *Aussiedler*/Turkish migrants are a burden for educational institutions in Germany,” “The German state should foster the cultural wealth (Children of) *Aussiedler*/Turkish migrants bring along,” and “The German state should secure that (Children of) *Aussiedler*/Turkish migrants have the same social rights like every other German.” Reliability was very good in all cases with alphas ranging between α = .82 and α = .84. Both item subsets were included to assess cognitive intolerance vis-à-vis outgroups as a latent variable. In addition to the attitudinal measure, the second measure assessed emotional *responses* and behavioral *reaction tendencies* towards the target groups. Four items assessed emotional responses. A sample item reads, “It makes me angry that *Aussiedler*/Turkish migrants live in Germany.” The other three items began with the phrases “It delights me…”, “It satisfies me…”, and “It worries me…”, respectively. Reliability was at α = .80, and thereby very good. Another four items measured behavioral intentions/tendencies: “I am fine with being friends with an Aussiedler/a Turkish migrant”, „I am fine with keeping company with *Aussiedler*/Turkish migrants”, “I would rather like to avoid *Aussiedler*/Turkish migrants”, and “I would like to oppose *Aussiedler*/Turkish migrants (turn against them)”. Reliability was at α = .88, and thereby excellent. Both the items on self-reported emotional responses and behavioral intentions were taken from Mackie, Devos, and Smith [48] in an adaptation by Hanke et al. [49]. Readers should note that we are not measuring actual self-reported behaviors, but behavioral intentions/tendencies, volitions in the terminology of Zhu [50]. Therefore, we use Zhu’s term ‘conative’ in the labelling of our latent variable that combines emotional reactions and behavioral intentions/tendencies.

Response options of all derogation items (attitudes as well as emotional responses and behavioral tendencies) ranged from ‘1’ (not at all) to ‘7’ (very much). The latent factors measuring attitudinal and conative-emotional intolerance were then combined into a second-order latent variable *outgroup derogation*.

#### Experimental manipulation

Participants were randomly assigned to only one set of attitude, emotion and behavior measures, either referring to diaspora migrants or Turkish migrants. For all included measures described above, we z-standardized scores within modes of data collection (paper-and-pencil vs. online) to avoid possible instrumentation artifacts. For a discussion of response style in mixed-mode studies see [51].

### Results and discussion

To test the two hypotheses of Study 1, we utilized a structural equation modeling (SEM) approach, using AMOS 24. In a first analysis, we tested whether the level of *TG* preferences indeed predicted outgroup derogation to a significantly different degree in the two experimental groups, i.e., among study participants who were asked for an evaluation of diaspora migrants and among study participants who were asked to evaluate Turkish migrants. In our analyses, we partialled all latent variables for gender and age of study participants, because these factors were not randomized between the two experimental groups so that group composition with regard to gender and age could have affected results under the two ‘treatments’ differently.

**Fig 1:**
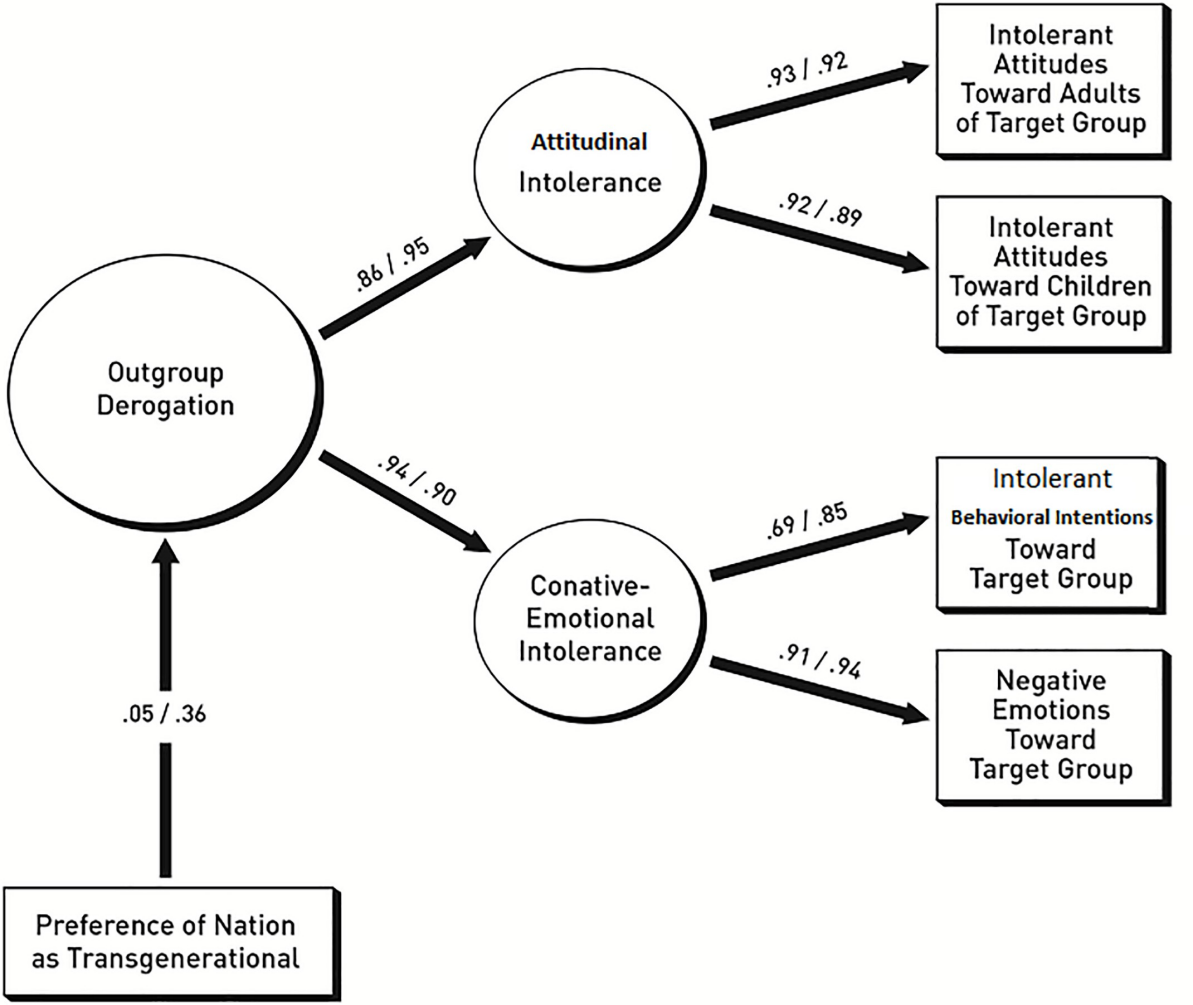
Prediction of derogation of diaspora migrants and of ‘foreign’ migrants by level of TG preferences. Left coefficient: Aussiedler/right coefficient: ‘foreign’ migrants.

Fig 1 documents the results of our SEM analyses, conducted as a two-group comparison. The graphic display of our results omits all error terms as well as the two control variables. Goodness-of-fit for the documented model was excellent: χ^2^ = 26.853, *df* = 23, *p* = .262, *CFI* = .993, *RMSEA* = .030, *PCLOSE* = .758. As shown in the figures the ß-coefficients from *TG* onto outgroup derogation differ substantially: For diaspora migrants the path is ß = .05, *p* = .661; for Turkish migrants the path is ß = .36, *p* < .001. Using Δ_χ_^2^ it was ascertained that the two coefficients differ significantly: The difference between the χ^2^ scores for a freely estimated model and a model restricting the path between *TG* and outgroup derogation to equality is Δ_χ_^2^ = 4.463, *p* = .035. Our finding confirms Hypothesis 1, namely that *TG* of study participants would predict derogation of outgroup members differently depending on whether they are diaspora or ‘foreign’ migrants.

Next, in order to test Hypothesis 2, we conducted three SEM analyses for the same model, in which we separately substituted *TG* by RWA, SDO, and HSI. As already stated in Footnote 6, we are *not* assuming that the four constructs measure uniquely different substance, to the contrary, we acknowledge that they largely overlap. It is not assumed that the non-overlap between *TG* and the other constructs is the unique component that drives the differential predictive effects on attitudes toward diaspora and foreign migrants. Instead, Hypothesis 2 suggests that these constructs in their intact entirety are related differentially to the derogation of diaspora as opposed to ‘foreign’ migrants. Table 1 offers details of these analyses in comparison to the analyses for *TG* as a predictor. Documented are standardized regression coefficients (ß) as well as Δ_χ_^2^ coefficients, which offer information on the χ^2^ goodness-of-fit for the freely estimated model as compared to a model where the path between predictor and criterion (outgroup derogation) is fixed to equality in both groups. A significant Δ_χ_^2^ indicates a significant difference in the size of the two ß coefficients to be compared.

**Table 1:** TG, RWA, SDO, and HSI as predictors of derogation of diaspora migrants as opposed to Turkish migrants.

	*TG*	RWA	SDO	HSI
**Target Diaspora Migrants**	.05	.52	.59	.38
**Target Turkish Migrants**	.36	.60	.60	.49
Δ_χ_^**2**^	4.463	1.232	0.894	1.139
***p***	.035	.267	.344	.286

*TG* = Perception of the group as Transgenerational; RWA = Right-Wing Authoritarianism; SDO = Social Dominance Orientation; HSI = Hierarchic Self-Interest

The table shows that all three alternative predictors explain more variance in outgroup derogation than did *TG* preferences. However, as indicated by the non-significant Δ_χ_^2^ scores, none of the three ‘competing’ concepts predicted derogation of diaspora migrants and of Turkish migrants differentially. Our findings, thus, also confirm Hypothesis 2 of Study 1, namely that only *TG*, but not other traditional predictors of outgroup derogation would predict derogation levels differentially for diaspora migrants as opposed to Turkish migrants. It came as a surprise to us, at the same time, that *TG* was not as powerful a predictor of outgroup derogation as were RWA, SDO, and HSI.

Study 1 offered initial evidence that the degree to which people conceive their nation as transgenerational—in a way eternal—does impact outgroup derogation. Other than people adhering to RWA, SDO, or HSI-affected attitudes, who do not distinguish in their outgroup derogation between migrants who are outgroup members by all plausible standards and outgroup members who share certain attributes with the ingroup, *TG* clearly predicts differential levels of derogation between diaspora migrants—who are not affected by significant derogation tendencies—and ‘foreign’ migrants who are derogated as expected.

## Study 2

The aim of Study 2 was again twofold: Firstly, we again tested Hypothesis 1, this time using a different methodological approach in order to evaluate the robustness of our findings. This time we used a measure of *positive* outgroup affect and behavior vis-à-vis diaspora and ‘foreign’ migrants (henceforth labelled outgroup appreciation) and also used a different experimental design. Again, we hypothesize that *TG* will be differentially related to the appreciation of the outgroup, depending on whether the evaluation target is a diaspora or a ‘foreign’ migrant. Secondly, in past research, *TG* had been found to correlate positively with identification with the nation [1] (*r* = .40). Hence, we test Hypothesis 3a, which states the relationship of *TG* and positive affect towards different outgroups persists after partialling for identification with the nation as such, and Hypothesis 3b stating that identification with the nation does not predict evaluations of diaspora migrants and ‘foreign’ migrants in different ways as *TG* does. A confirmation of Hypotheses 3a and b would suggest that nationalism as such is not the source of distinguishing between different outgroups.

### Method

#### Participants

Study 2 was conducted among 235 non-immigrant German adults (41.6% female, mean age; 40.9, ranging from 16 to 76.). To obtain a heterogeneous sample, multiple modes of data acquisition were once again employed. Data were collected via an online survey (N = 113) again distributed through various web-based forums and online social networks, and via distributing paper versions of the survey instrument (N = 122) to a varied audience (an adult education center in the Bremen area, among university students, and in various neighborhood places, like barber shops and church gathering places). As in Study 1, participants of Study 2 had a high level of education (more than one third reported holding a university degree), expressed low levels of religiosity (more than a third declared to ‘not at all’ be religious, whereas only six percent saw themselves as very religious); in their political preferences participants of Study 2 were on average a bit more conservative than those of Study 1 (again 57% saw themselves as left or liberal, but this time only 18 percent saw themselves as moderates, whereas 25% saw themselves as conservative or right-wing).

#### Measures

Participants were randomly exposed to two different conditions in a between-subjects design. After completing the *TG* measure, they received a description of a person who had won in a lottery of a local newspaper and was interviewed by the newspaper. The text (presented as a newspaper article) portrayed the target person. An English version is included in the Appendix for Study 2. All information was identical in both conditions, except for the fact that the winner was either a diaspora migrant or a Turkish migrant.

As in Study 1, all included measures described above were z-standardized within the sub-samples studied via different modes of data collection (paper-and-pencil vs. online) to avoid possible instrumentation artifacts.

For the assessment of *TG*, the same four-item scale as in Study 1 was used. Scale consistency this time was at α = .81. Outgroup appreciation was again measured comprehensively using a combination of measures of (positive) emotional reactions as well as (positive) behavioral tendencies towards the target person (diaspora vs. ‘foreign’ migrants). Four items measured positive emotional reactions. The instruction read, “Please recall the person described [in the experimental manipulation]. Which feelings do you have towards this person? When I think about this person, I feel …” followed by the respective emotion “pleased”, “satisfied”, “cheerful”, and “happy”, respectively (α = .66). Positive behavioral tendencies were measured by four items. Each item started with the phrase “I would be fine with…” followed by the phrases “helping this person if needed”, “keeping company with this person”, “working together with this person”, and “being friends with this person” (α = .90). The emotion and behavior items once again were taken from Mackie, Devos, and Smith [48] in their adaptation by Hanke et al. [49]. For all items, response categories ranged from ‘1’ (totally disagree) to ‘7’ (totally agree). The items were combined to a latent factor *Outgroup Appreciation*. A 16-item measure developed by Roccas, Klar, and Liviatan [52] was included to measure *identification with the nation*. This scale consisted of four sub-dimensions, measuring subjective importance of the nation (e.g., “To belong to the Germans—as a group—is an important part of my identity”) and commitment to it (e.g., “I have a strong sense of obligation towards the Germans—as a group”), as well as perceived superiority of (e.g., “Other groups can learn a lot from us Germans”) and deference to one’s nation (e.g., “In difficult times the only way to know what to do is to rely on the leaders of this country”). Consistency of the overall scale was very high (α = .94).

### Results and discussion

In Step 1, the analytic strategy of Study 2 resembled the one utilized in Study 1. In a first analysis, we tested whether the level of *TG* preferences indeed predicted outgroup appreciation to a significantly different degree in the two experimental groups, i.e., among study participants who were asked for an evaluation of diaspora migrants and among study participants who were asked to evaluate Turkish migrants. In our analyses, we again controlled for gender and age of study participants.

**Fig 2:**
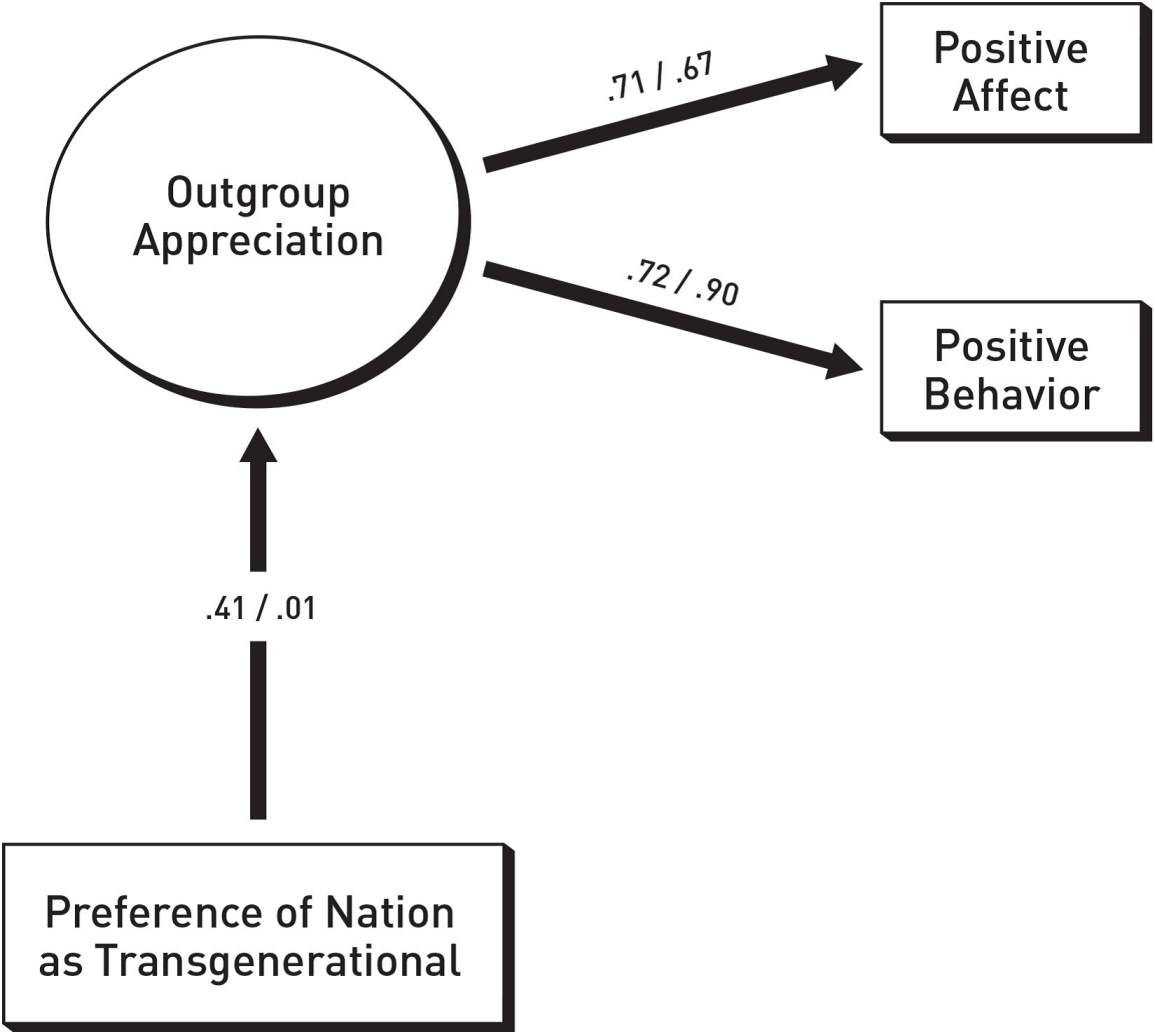
Prediction of appreciation of diaspora migrants and of ‘foreign’ migrants by level of TG preferences. Left coefficient: diaspora migrants/right coefficient: ‘foreign’ migrants.

Fig 2 documents the results of the SEM analyses, conducted as a two-group comparison. The graphic display of our results once again omits all error terms as well as the two control variables. Goodness-of-fit for the documented model was excellent: χ^2^ = 7.250, *df* = 5, *p* = .203, *CFI* = .979, *RMSEA* = .044, *PCLOSE* = .483. As shown in the figures, the ß-coefficients from *TG* onto outgroup appreciation differ substantially: For diaspora migrants the path is ß = .41, *p* < .001; for Turkish migrants the path is ß = .01, *p* = .887. Using Δ_χ_^2^ it is ascertained that the two coefficients differ significantly from each other: The difference between the χ^2^ scores for the freely estimated model and the model restricting the path between *TG* and outgroup appreciation to equality is Δ_χ_^2^ = 4.914, *p* = .027. Our finding again confirms Hypothesis 1, namely that *TG* of study participants predicts appreciation of outgroup members differently depending on whether they are diaspora or ‘foreign’ migrants. It should be noted that in Study 2, *TG* positively predicted appreciation of the diaspora migrant outgroup, whereas appreciation of Turkish migrants was not related significantly to *TG*.

Next, we conducted an SEM analysis, which included *identification with the nation* as a (further—beyond gender and age) covariate. The purpose of this analysis was to check whether the differential consequences of the level of *TG* for the appreciation of different target groups (diaspora and Turkish migrants) is a consequence of the fact that *TG* might just be another variant for measuring identification with one’s nation. Unlike in Study 1, where the contrast variables were by and large variables measuring personality dispositions, here our contrast variable is conceptually very closely related to TG in its reference to the entity ‘nation.’ Thus, unlike in Study 1, a test is needed as to whether the two constructs indeed have sufficiently different contents. When the additional covariate was introduced into the model the ß coefficients from *TG* onto outgroup appreciation did not change much. They are now at ß = .46, *p* < .001 (diaspora migrants), and at ß = .12, *p* = .292 (Turkish immigrants). The difference was still significant: Δ_χ_^2^ = 4.471, *p* = .034. *TG* continued to predict the appreciation of diaspora migrants positively, whereas it did not contribute significantly to the prediction of appreciation of Turkish migrants. Identification with the nation predicted outgroup appreciation negatively for diaspora migrants (ß = -.16, *p* = .173) and for Turkish migrants (ß = -.29, *p* = .038), but only for Turkish immigrants was the path significant. The Δ_χ_^2^ = 0.302, *p* = .583 revealed, however, that the two coefficients do not differ significantly between the two groups. It can, therefore, be concluded that *TG* and *identification with the nation* are not variables that measure in principle the same psychological substance. Hypotheses 3a and 3b of Study 2 are thus also confirmed.

The most notable finding of Study 2 is that whereas in Study 1 high levels of *TG* enhanced levels of derogation of ‘foreign’ migrants while not affecting levels of derogation of diaspora migrants, in Study 2 high levels of *TG increased* appreciation of diaspora migrants, whereas appreciation of Turkish migrants was not affected. This finding indicates that perceiving one’s nation as a transgenerational entity indeed affects outgroup derogation and outgroup appreciation differently. *TG* enhances derogation levels of ‘foreign’ outgroups, while at the same time enhancing appreciation of diaspora migrants, who are to some degree ‘of our kind.’ It has also been shown that *TG* is not the same as nationalism: Identification with one’s nation dampens the appreciation of outgroups *regardless* of their ethnic belonging. We will follow up on these results by testing if *TG* affects the explicit acceptance of different groups of migrants as citizens of Germany differently.

## Study 3

In Study 3, we also tested Hypothesis 1, this time using yet another measure of group evaluation, namely the explicit acceptance of different groups of migrants as citizens of Germany. Our hypothesis is that again *TG* will differentially predict the number of criteria to be fulfilled by diaspora as opposed to ‘foreign’ migrants to gain German citizenship (see Hypothesis 1). Specifically, we assume that people who express high *TG* require ‘foreign’ migrants to fulfill more criteria for citizenship than diaspora migrants.

### Method

Study 3 was conducted among 232 non-immigrant German adults (49.6% female; mean age: 23.1, ranging from 19 to 65.). This time all participants were university students (attendees of an undergraduate sociology lecture at Universität Bremen, the local public university). As in Study 1 and 2, participants of Study 3 expressed low levels of religiosity (one-third declared to ‘not at all’ be religious, whereas only three percent saw themselves as very religious); in their political preferences participants leaned towards the left as did the other two samples (55% saw themselves as left or liberal, 26% saw themselves as moderates, whereas 19% saw themselves as conservative or right-wing).

#### Measures

For the assessment of *TG*, the same four-item scale that was used as in Study 1 and Study 2. Scale consistency this time was at α = .75. Nine items were used to quantify the *degree of exclusionism*. The collection of items was not meant as a psychometric scale, but as a formative index [53]. It follows the ‘basket of goods’ logic of consumer research; people ‘shop’ from an available list of goods. The overall number (sum) of the chosen goods then constitutes the score of the variable. In our case, people had to tick items that they thought migrants would have to fulfill to be eligible to become German citizens. The nine items were, “They have to reside in Germany,” “Their center of vital interests has to be in Germany,” “They have parents who were born in Germany,” “They do not have a criminal record,” “They accept federal laws and regulations,” “They advocate the liberal-democratic constitutional order of Germany,” “They adjust to ways of life common in German society,” “They came to Germany to settle here permanently,” and “They do not partake in criminal activities.” Each of these items could either be ticked or not ticked. Empirically, scores varied between 0 and 9, with a mean, median, and mode of 5. The list was a sub-selection of items developed by the Israeli part of the research team. The formative index, i.e., the sum of all ticked items was used as a manifest variable in the subsequent analyses.

#### Experimental manipulation

Participants were randomly assigned to one of two questionnaire versions, namely one that asked respondents to tick items of the exclusionism battery for Turkish migrants, and one that asked respondents to tick items of the exclusionism battery for diaspora migrants. As all data were obtained via paper-pencil instruments in Study 3, no standardization of scores was performed before proceeding with the analyses.

### Results and discussion

Also in this case, our analyses were conducted in the SEM framework. In a two-group SEM comparison, we tested whether the level of *TG* indeed predicted the manifest variable ‘degree of exclusionism’ to a significantly different degree in the two experimental groups, i.e., among study participants who were asked for criteria they thought diaspora migrants should fulfill to gain citizenship and among study participants who were asked for the number of requirements for citizenship, Turkish migrants should fulfill. Again, we partialled for age and gender.

The tested model as such was a saturated model with *df* = 0, *CFI* = 1.000, *RMSEA* = .038, *PCLOSE* = .734. However, by fixing the ß-coefficient from *TG* onto outgroup exclusion to equality between the two groups of migrants, one gains one degree of freedom. Using Δ_χ_^2^ (with df = 1), one can then test whether coefficients for diaspora migrants (ß = -.11, *p* = .170) and for Turkish migrants (ß = .20, *p* = .010) differ significantly. The difference between the χ^2^ scores for the freely estimated model and the model restricting the path between *TG* and outgroup appreciation to equality is Δ_χ_^2^ = 7.756, *p* = .005. Hence, also Study 3 confirms Hypothesis 1 that *TG* of study participant would predict exclusionism with regard to citizenship eligibility differently depending on whether outgroup members are diaspora or ‘foreign’ migrants.no diff

Once again, however, effect sizes were small; only about 4% of the variance in exclusionism vis-à-vis ‘foreign’ migrants were explained by *TG*. The core finding also of Study 3 was that people who see their own national group as a transgenerational entity (rather than as an entity comprising only the current generation of compatriots) distinguish in their evaluative statements about outgroups (here about requirements outgroup members have to fulfill before they become part of the ingroup) along the lines of the symbolic ingroup-outgroup overlap.

## Ethics statement

All procedures performed in studies involving human participants were in accordance with the ethical standards of the institutional and/or national research committee and with the 1964 Helsinki declaration and its later amendments or comparable ethical standards. Informed consent was obtained prior to participation from all individual participants included in the study. All data collection procedures were peer-reviewed ahead of their enactment by the funder of the study, the German-Israeli Foundation for Scientific Research and Development (GIF). GIF issued no specific additional ethics requirement with its granting letter. Data collection took place during the years 2012 to 2015 before the enactment of the EU data protection guidelines in May 2018, however, it complied with all regulations detailed in these guidelines. The collected data were fully anonymized prior to the analyses and did not contain any personal information of participants. The Ethics Committee of Jacobs University Bremen has been informed about the study upon submission of the manuscript and voiced no concerns.

## General discussion

The presented studies find that the incorporation of a time dimension in the boundaries of one’s national ingroup definition indeed has implications for the way in which outgroups are perceived. Specifically, we find that people who perceive their own national ingroup as transgenerational express more derogatory evaluations vis-à-vis ‘foreign’ than vis-à-vis diaspora migrants. In and by itself, this finding would probably not be a major advance in nationalism research. However, it gains importance through the finding that whereas *TG* does predict outgroup derogation differentially for diaspora and ‘foreign’ migrants, traditional predictors of outgroup derogation (right-wing authoritarianism, RWA: [35]; social dominance orientation, SDO: [36]; hierarchic self-interest, HSI: [37]) *do not*. This means that *TG* is not just another personality proxy for outgroup derogation tendencies, but captures an aspect of nationalism that is not covered by other variants of right-wing and related orientations. One must at the same time concede that *TG* is not a strong predictor of outgroup derogation in and by itself: The power of RWA, SDO, and HSI in predicting outgroup derogation is considerably higher. *TG*’s conceptual value lies in its ability to differentially predict derogation of outgroups that differ in their otherness in comparison to the national ingroup.

Secondly—in Study 2—we find that *TG* not only differentially predicts derogation of diaspora as opposed to ‘foreign’ migrants, but that it also differentially predicts appreciation of ‘foreign’ as opposed to diaspora migrants. Whereas for derogation strong *TG* is positively related to the derogation of ‘foreign’ migrants and unrelated to derogation of diaspora migrants, the picture is reversed for outgroup appreciation: High *TG* positively predicts appreciation of diaspora migrants, while being unrelated to appreciation of Turkish migrants. Lastly, we find that a perception of the national group as *TG* differentially affects the number of citizenship criteria that diaspora as opposed to ‘foreign’ migrants are expected to fulfill.

This research is one of the first to test how the temporal inclusiveness of the ingroup relates to attitudes towards outgroups. The findings suggest that *TG* allows a more fine-tuned assessment of attitudes towards different types of migrants than do common predictors of nationalistic outgroup derogation. This is an important step to understand the complex nature of collective identity.

The fact that the differential effects of *TG* on outgroup evaluations can be found across different study designs and evaluation measures points to the robustness of the findings. They are in accordance with previous studies which found that the incorporation of a temporal dimension in one’s concept of national identity is associated with a higher rejection of national outgroups ([54] with regard to collective nostalgia; [7] as well as [55] for collective self-continuity). Yet, the current study further differentiates these negative effects on outgroup attitudes with regard to different types of ‘others.’

Although the explained variances observed in the presented studies were relatively low, the findings suggest that *TG* is a relevant construct to include in the assessment of intergroup attitudes in contemporary societies. In the context of an influx of new ‘foreign’ migrants to many European countries due to the Syrian civil war, and to political and economic crises in Afghanistan, Iraq, and various parts of Africa, it is important to consider how people’s concepts of their national group affects reactions of the receiving societies, and whether or not these concepts include a temporal dimension that shapes outgroup attitudes. In particular, if a nation puts too much emphasis on its transgenerational nature, this will potentially result in more restrictive immigration regulations for those who ‘do not belong’ to this nation. From a humanistic and cosmopolitan point of view, a nation should be conceded to include past generations in its self-understanding, but it should also acknowledge that the current generation can undergo changes (e.g., through new members) which then shape future generations. Or in line with Hannah Arendt’s credo [56]: every human being is per se a transgenerational entity, whereas nations come and go. This calls for policies that directly target exclusionary views on national belonging, but also on other psychological and sociological factors that underlie such views, such as political ideologies, exclusionary religious beliefs, or authoritarian world views.

To gain yet more clarity about the psychological importance of *TG* for nationalism research, we see a necessity to empirically differentiate further the nomological network of the *TG* concept from other concepts that incorporate a temporal dimension to the ingroup definition to prevent conceptual ambiguity. For example, there might be a conceptual overlap between *TG* and an ethnic definition of nationhood (at least in the German context): Individuals high on *TG* view diaspora migrants more positively simply due to their ethnic descent. What speaks against this argument is that the German population generally perceives diaspora migrants, although of ethnic German descent, as immigrants (‘Russians’) who compete over resources in the society, and thus negative attitudes towards them are rather prevalent [36,39,30,53]. Hence, simply being an immigrant of German descent does not necessarily lead to being accepted by the host population. However, the conceptual overlap of *TG* and an ethnic definition of nationhood needs further attention in future studies. We explored this potential overlap with data we collected in a pilot study (not reported here, N = 161). In that sample the *TG* scale is unrelated to an item that assesses an ethnic definition of nationhood (“People should be granted the German citizenship if they have German ancestors, e.g. parents, grandparents or great grandparents”). However, albeit none of the coefficients are significant, the correlations between this item and outgroup attitudes show a similar pattern like *TG* (positive coefficients for attitudes towards diaspora migrants and negative coefficients for attitudes towards Turkish immigrants). One possibility is to control for ethnic conceptions of nationhood in future studies. Alternatively, the assumptions of the current paper could be tested with a group that has been living in the area of today’s Germany for centuries but is not ethnically Germans. There is indeed a minority group in Germany that meets this criterion, the Sorbs, a minority group of Slavic descent [e.g., 57]. Analogous to the current study, the assumption would be that *TG* is related to the derogation of Turkish immigrants but not to the derogation of Sorbs.

In a similar vein, one could argue that, since *TG* also involves future generations of one’s nation, individuals high on *TG* should also accept current ‘foreign’ immigrants (such as Turkish immigrants) because these are viewed as part of future generations. We assume that this depends on how much an individual is generally willing to accept recent newcomers (e.g., Turkish immigrants) as future ingroup members. Such a moderator function of ‘inclusiveness’ could be yet another focus of future research.

From a methodological perspective, we compared the role of *TG* for outgroup evaluations with the role of other relevant theoretical constructs such as RWA but deliberately did not simultaneously include these other constructs in all studies. It might be advisable to do so after all in order to further sharpen the *TG* concept vis-á-vis other relevant construct in a study with a much larger sample size in the future.

Apart from these conceptual considerations, the choice of ethnic German diaspora immigrants and Turkish immigrants as target groups might have had an impact on how *TG* relates to attitudes towards these groups. As we described in the introduction, both groups indeed differ with regard to their societal status, however, they also show striking similarities. Nevertheless, it cannot be ruled out that different effects of *TG* are at least partly due to different characteristics of these groups. As already argued above, future studies should therefore investigate *TG* with regard to attitudes towards other groups as well.

Finally, although our findings have policy implications (as outlined above), a generalization towards the German population as a whole is not advisable since sampling strategies were not guided by random probability sampling and the sample sizes of our studies were not overly large. The current study attempted to demonstrate the general psychological implications of including a temporal dimension in one’s conceptualization of nationhood. What is worth researching within this framework is the role national contexts play, an issue we somewhat neglected in the current study. Germany has a particular way of dealing with one’s national past. The National Socialist German Workers Party (NSDAP) which came into office in 1933 had organized the holocaust, an incredibly horrible genocide that cost the lives of around six million Jews and that impacts public narratives on nationhood in Germany until today. Postwar governments in the divided Germany initially distanced themselves from this past by pushing ‘not us’ narratives in the communist East and by suppressing any German history references in public narratives in the West that concerned the time after 1930. This led to any mention of ‘the nation’ becoming a no-go-area in mainstream discourse until 1990, the year of German unification. Only since then ‘the nation’ did once again become a debatable, but nevertheless skeptically looked-upon concept in mainstream politics. An illustration of the controversial nature of the concept of the nation in Germany and its role in the political sphere lies in the later-year biography of conservative Chancellor Helmut Kohl who advocated an understanding of ‘the nation’ as a part of a united Europe. When he died in July 2017, his last will denied the organization of a German state funeral and instead called for one that would honor him as an Honorary Citizen of Europe. The funeral took place in Strasbourg, outside Germany, but within Europe. Interestingly, this struggle with a historical sense of identification with the nation contradicts with the practice of naturalizing ethnic Germans on the basis of their ancestral ties. Hence, in order to shed light on the role of national contexts for *TG*, it is important to conduct studies simultaneously in several national contexts. For example, post-colonial nations such as the United Kingdom and the Netherlands also have diaspora migrants (in the sense of residents of former colonies) and ‘foreign’ migrants (in the context of work migration), but look back to different historical pasts. Moreover, in contrast to the German context, migrants from former colonial territories are likely to be of different ethnicities, which is not the case for the German diaspora migrants. Future research should, therefore, assess if ethnicity moderates the effect of *TG* on differential attitudes towards different groups of migrants.
